# CoFe_2_O_4_@MIL-100(Fe) hybrid magnetic nanoparticles exhibit fast and selective adsorption of arsenic with high adsorption capacity

**DOI:** 10.1038/srep40955

**Published:** 2017-01-19

**Authors:** Ji-Chun Yang, Xue-Bo Yin

**Affiliations:** 1 State Key Laboratory of Medicinal Chemical Biology and Tianjin Key Laboratory of Biosensing and Molecular Recognition, College of Chemistry, Nankai University, Tianjin, 300071, China; 2 Collaborative Innovation Center of Chemical Science and Engineering (Tianjin), Nankai University, Tianjin, 300071, China

## Abstract

In this study, we report the synthesis and application of mesoporous CoFe_2_O_4_@MIL-100(Fe) hybrid magnetic nanoparticles (MNPs) for the simultaneous removal of inorganic arsenic (iAs). The hybrid adsorbent had a core-shell and mesoporous structure with an average diameter of 260 nm. The nanoscale size and mesoporous character impart a fast adsorption rate and high adsorption capacity for iAs. In total, 0.1 mg L^−1^ As(V) and As(III) could be adsorbed within 2 min, and the maximum adsorption capacities were 114.8 mg g^−1^ for As(V) and 143.6 mg g^−1^ for As(III), higher than most previously reported adsorbents. The anti-interference capacity for iAs adsorption was improved by the electrostatic repulsion and size exclusion effects of the MIL-100(Fe) shell, which also decreased the zero-charge point of the hybrid absorbent for a broad pH adsorption range. The adsorption mechanisms of iAs on the MNPs are proposed. An Fe-O-As structure was formed on CoFe_2_O_4_@MIL-100(Fe) through hydroxyl substitution with the deprotonated iAs species. Monolayer adsorption of As(V) was observed, while hydrogen bonding led to the multi-layer adsorption of neutral As(III) for its high adsorption capacity. The high efficiency and the excellent pH- and interference-tolerance capacities of CoFe_2_O_4_@MIL-100(Fe) allowed effective iAs removal from natural water samples, as validated with batch magnetic separation mode and a portable filtration strategy.

Arsenic is one of the most important contaminants with high toxicity in natural water systems worldwide. In addition to anthropogenic release, naturally occurring pathways from As-containing soil, minerals, and ores are also important pollution sources of inorganic arsenic (iAs), including arsenate [As(V)] and arsenite [As(III)]^1^,^2^,^3^,^4^. More than 226 million people have been affected by arsenic pollution around the world^5^. A significant positive correlation was observed between water As concentration and As content in urine, nail, and hair samples, as well as arsenicosis, such as severe skin lesions^6^. Long-term exposure to an As-contaminated environment causes cancer, dermatitis, respiratory diseases, neurotoxicity, and even death^7^,^8^. The World Health Organization (WHO) has therefore established a maximum-allowed-concentration of 10 μg L^−1^ iAs in drinking water to minimize the health risk to human beings^9^,^10^. Therefore, the effective and efficient removal of iAs from natural water samples has raised worldwide public concern.

The adsorption method is well suited for the batch-treatment of natural water samples as the simplest, most cost-effective and user-friendly technology for iAs removal^11^,^12^. Ferrous magnetic nanoparticles (MNPs) are outstanding adsorbents for iAs removal because of the strong and irreversible interaction between iAs and iron oxide^13^,^14^. Superparamagnetic Fe_3_O_4_, Fe_3_O_4_-graphene composites, hematite-coated Fe_3_O_4_, and polystyrene-supported nano-Fe_3_O_4_ have been exploited to remove iAs from water samples^15^,^16^,^17^,^18^. However, MNP adsorbents usually suffer from low adsorption capacity, slow adsorption kinetics^19^,^20^, and low pH- and interference-tolerance capacities^21^,^22^. In addition, soluble iron may increase the toxicity of iAs. Thus, various TiO_2_-based adsorbents have been developed^9^,^23^,^24^,^25^. However, magnetic separation and the strong interaction of Fe-O-As are still highly attractive merits of magnetic adsorbents. Improving the effectiveness and stability of MNP adsorbents for iAs with high adsorption efficiency by a simple treatment procedure is therefore critically required.

The other problem is the confusion regarding the adsorption mechanism of iAs on ferrous magnetic materials. Some works confirmed the selective adsorption of As(V) but low adsorption efficiencies for As(III)^26^,^27^,^28^,^29^,^30^; other works showed high adsorption capacities toward As(III)^19^,^31^,^32^. Clear evidence was illustrated for an inner-sphere complexation mechanism, but the nature of the surface complexes was controversial for the adsorption of As(V) and As(III)^6^.

One of the strategies to improve adsorption capacity is to increase the adsorption surface area and the active sites of the adsorbents^33^. Decreasing the magnetite size is one of the options. Colvin *et al*.^14^ reported more than 150 mg g^−1^ of maximum As adsorption capacity with 12-nm magnetite nanocrystals, but the nanocrystals aggregated easily. Mesoporous magnetic adsorbents are also an alternative to improve the adsorption capacity because of their high specific surface area and uniform and tunable pore size compared to Fe_3_O_4_ MNPs^21^,^34^,^35^,^36^. Bimetal oxide MNPs present the advantage and synergistic effects of the parent metal oxides and provide abundant oxygen-containing functional groups^34^,^35^,^36^. However, bimetal oxide MNPs also showed high chemical reactivity and agglomeration. Applying surface functionalization and coatings to the MNPs would overcome these challenges^37^,^38^.

The selectivity and stability of the adsorbent could also be improved by coating porous materials on the surface of the MNPs. Metal-organic frameworks (MOFs) are crystalline materials constructed from metal ions or clusters and organic ligands. Their unique porosity, stability, and versatility^39^,^40^,^41^ make MOFs ideal for pollutant removal, including iAs^42^. MIL-100(Fe) is one kind of MOF built with benzene-1,3,5-tricarboxylate (H_3_BTC) and iron trimeric octahedral clusters with permanent pores^43^,^44^.

Herein, we report the synthesis and application of a core-shell CoFe_2_O_4_@MIL-100(Fe) hybrid material as an effective, stable, and efficient mesoporous magnetic adsorbent for the simultaneous removal of iAs. A rapid uptake rate (0.1 mg L^−1^ iAs could be adsorbed within 2 min) and high adsorption capacity [114.8 mg g^−1^ for As(V) and 143.6 mg g^−1^ for As(III)] was observed because of its nanoscale size and mesoporous properties. An excellent anti-interference capacity was confirmed by using the electrostatic repulsion interaction and size exclusion effect of the MIL-100(Fe) shell. MIL-100(Fe) also decreased the zero-charge point (ZCP) of the hybrid absorbent to improve iAs adsorption over the wide pH range of 4–10. The adsorption kinetics, isotherms, and thermodynamics of iAs on the hybrid adsorbent were carefully studied to propose the adsorption mechanism of iAs. Both As(V) and As(III) were adsorbed to form the Fe-O-As microstructure with the inner-sphere complex mechanism, although As(V) and As(III) showed monolayer and multilayer adsorption, respectively. Their differential adsorption behaviors were controlled by the dissociation constants of arsenate and arsenite. Two kinds of simple water treatment strategies were proposed to illustrate the practicability of the hybrid absorbent for iAs removal in natural water samples: a batch mode with simple magnetic separation and a filtration strategy for the simultaneous removal of solid particles and iAs. To our surprise, we found that MIL-100(Fe) only showed favorable adsorption of As(V). CoFe_2_O_4_@MIL-100(Fe) illustrated a high adsorption capacity toward both As(V) and As(III) to determine the total iAs content. The difference between the two results was therefore used to calculated the content of As(III). We found high As(III) content in rural well water from Shanxi, China, a typical sample of hypoxic As-contaminated groundwater.

## Results and Discussion

### Preparation and characterization of the hybrid adsorbents

**Figure 1:**
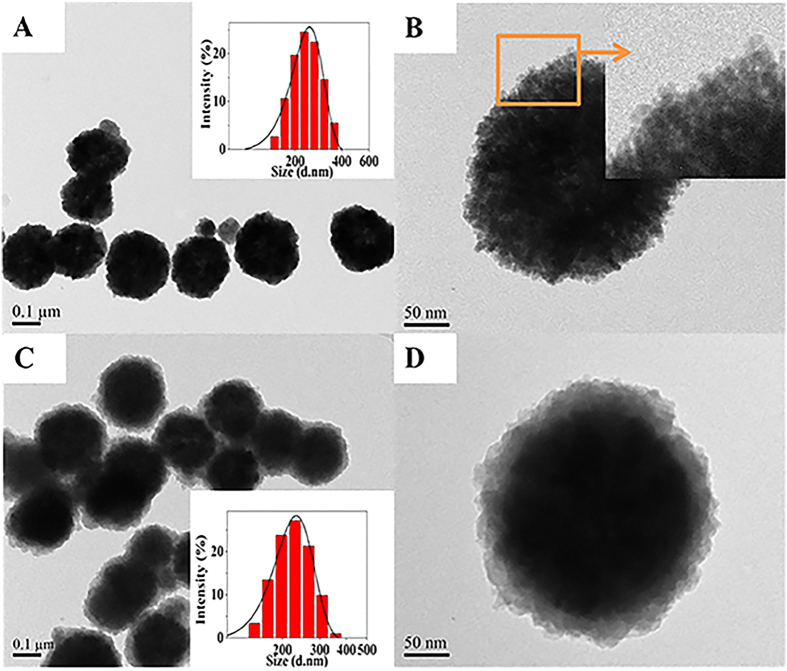
Morphology of CoFe_2_O_4_ and CoFe_2_O_4_@MIL-100(Fe). TEM images of (**A**,**B**) CoFe_2_O_4_ and (**C**,**D**) CoFe_2_O_4_@MIL-100(Fe); inset: size distributions from dynamic light scattering. The TEM images indicate that both CoFe_2_O_4_ and CoFe_2_O_4_@MIL-100(Fe) MNPs exhibited excellent nanoscale and mesoporous properties.

Mesoporous CoFe_2_O_4_ magnetic nanoparticles (MNPs) were synthesized by a facile one-pot hydrothermal treatment of CoCl_2_, FeCl_3_, CH_3_COONa, and PEG-6000. CoFe_2_O_4_@MIL-100(Fe) hybrid MNPs were then prepared by a step-by-step self-assembly strategy. Transmission electron microscopic (TEM) images of CoFe_2_O_4_ and CoFe_2_O_4_@MIL-100(Fe) MNPs clearly illustrated their spherical structure and porosity, with average diameters of 225 and 260 nm, respectively (Fig. 1). Moreover, the uniform MIL-100(Fe) layer with a thickness of ca. 18 nm was successfully coated onto the surface of CoFe_2_O_4_ to form the CoFe_2_O_4_@MIL-100(Fe) hybrid adsorbent with a core-shell microstructure. Dynamic light scattering analysis revealed that CoFe_2_O_4_ and CoFe_2_O_4_@MIL-100(Fe) MNPs had relatively narrow size distributions and were well dispersed for real applications (insets in Fig. 1A and C). Fast diffusion kinetics and high adsorption capacities are expected for iAs adsorption based on the nanoscale size and mesoporous properties of CoFe_2_O_4_@MIL-100(Fe) compared with its bulk counterpart.

**Figure 2:**
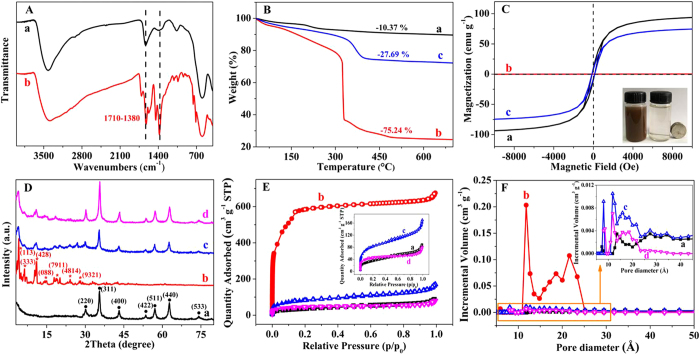
Characterization of CoFe_2_O_4_ and CoFe_2_O_4_@MIL-100(Fe). (**A**) FTIR spectra of (a) CoFe_2_O_4_ and (b) CoFe_2_O_4_@MIL-100(Fe); (**B**) TGA and (**C**) magnetic hysteresis curves of (a) CoFe_2_O_4_, (b) MIL-100(Fe), and (c) CoFe_2_O_4_@MIL-100(Fe); (**D**) XRD patterns, (**E**) N_2_ adsorption-desorption isotherms, and (**F**) DFT pore size distributions of (a) CoFe_2_O_4_, (b) MIL-100(Fe), (c) CoFe_2_O_4_@MIL-100(Fe), and (d) CoFe_2_O_4_@MIL-100(Fe) after the adsorption of As(V) [10 mL of 100 mg L^−1^ As(V) solution treated with 5 mg of the adsorbent]. All of the characterizations demonstrated that the MIL-100(Fe) shell was successfully coated on the surface of CoFe_2_O_4_ and that the hybrid adsorbent was highly stable.

The comparison of the Fourier transform infrared spectra (FTIR) between CoFe_2_O_4_ and CoFe_2_O_4_@MIL-100(Fe) MNPs confirmed that MIL-100(Fe) has been successfully introduced into the hybrid magnetic material (Fig. 2A). After coating with MIL-100(Fe), peaks appearing between 1710 cm^−1^ and 1380 cm^−1^ were assigned to the typical adsorption of the organic ligand (H_3_BTC). Thermogravimetric analysis (TGA) results of CoFe_2_O_4_, MIL-100(Fe), and CoFe_2_O_4_@MIL-100(Fe) revealed that CoFe_2_O_4_ showed high stability in the tested temperature range (Fig. 2B). The gradual weight loss before 300 °C was attributed to the removal of the solvents ethylene glycol and water from both MIL-100(Fe) and CoFe_2_O_4_@MIL-100(Fe). The significant and fast weight loss occurring at 330 °C was assigned to the collapse of the MIL-100(Fe) skeleton upon the decomposition of H_3_BTC.

The magnetic properties of CoFe_2_O_4_, MIL-100(Fe), and CoFe_2_O_4_@MIL-100(Fe) were investigated at room temperature by a Vibrating Sample Magnetometer (VSM) applying a field of ±10 kOe (Fig. 2C). Their magnetic hysteresis curves illustrated that CoFe_2_O_4_ and CoFe_2_O_4_@MIL-100(Fe) showed typical soft ferromagnetism, while MIL-100(Fe) was non-magnetic. The specific saturation magnetization (*M*_*s*_) of CoFe_2_O_4_ decreased from 102.3 to 81.4 emu g^−1^ after it was integrated with the non-magnetic MIL-100(Fe). However, the magnetization value of 81.4 emu g^−1^ is still considerable and sufficient to collect the hybrid MNPs from the solution by a magnet (inset of Fig. 2C).

Powder X-ray diffraction (XRD) patterns of CoFe_2_O_4_, MIL-100(Fe), and CoFe_2_O_4_@MIL-100(Fe) were recorded (Fig. 2D). Peaks observed at 30.4, 35.7, 43.4, 53.8, 57.3, 62.7, and 74.7° were assigned to the (220), (311), (400), (422), (511), (440), and (533) planes of spinel CoFe_2_O_4_ (JCPDS No. 22–1086). The peaks of MIL-100(Fe) at 2*θ* values of 3.9, 5.3, 11, 14.2, 18.2, 20.1, and 27.7° correspond to the (113), (333), (428), (088), (7911), (4814), and (9321) planes of crystalline MIL-100(Fe)^45^. The PXRD pattern of the fresh CoFe_2_O_4_@MIL-100(Fe) hybrid MNPs matches well with those of both cubic spinel phase CoFe_2_O_4_ and crystalline MIL-100(Fe) (Fig. 2D). Moreover, the XRD pattern of CoFe_2_O_4_@MIL-100(Fe) remained unchanged after the adsorption of As(V), suggesting that As(V) was only adsorbed on the inner and outer surface to maintain the crystal structure. Thus, CoFe_2_O_4_@MIL-100(Fe) is highly stable as an iAs adsorbent.

The surface area and pore size distribution, which significantly influence the adsorption capacity, are essential properties for iAs adsorbents. The Brunauer-Emmett-Teller (BET) surface areas of CoFe_2_O_4_, MIL-100(Fe), and CoFe_2_O_4_@MIL-100(Fe) were measured as 127, 2109, and 292 m^2^ g^−1^ by N_2_ adsorption/desorption isotherms (Fig. 2E). Pore size distributions determined by the DFT method give the pore diameters of 47.2, 1.0, and 20.6 nm with the pore volumes of 0.09, 0.9, and 0.16 cm^3^ g^−1^ for CoFe_2_O_4_, MIL-100(Fe), and CoFe_2_O_4_@MIL-100(Fe), respectively (Fig. 2F). The porous structure and large surface area make the CoFe_2_O_4_@MIL-100(Fe) hybrid material ideal as an adsorbent for iAs removal. The 1.0 nm micropores of the MIL-100(Fe) shell actually show a molecular-sieving effect as a restricted-access coating. The BET surface area and pore volume of CoFe_2_O_4_@MIL-100(Fe) decreased from 292 m^2^ g^−1^ to 153 m^2^ g^−1^ and from 0.16 to 0.07 cm^3^ g^−1^ after the adsorption of As(V), suggesting that As(V) adsorbed onto both the surface and interior of the hybrid MNPs adsorbent.

### Effect of pH on iAs adsorption on the hybrid adsorbent

**Figure 3:**
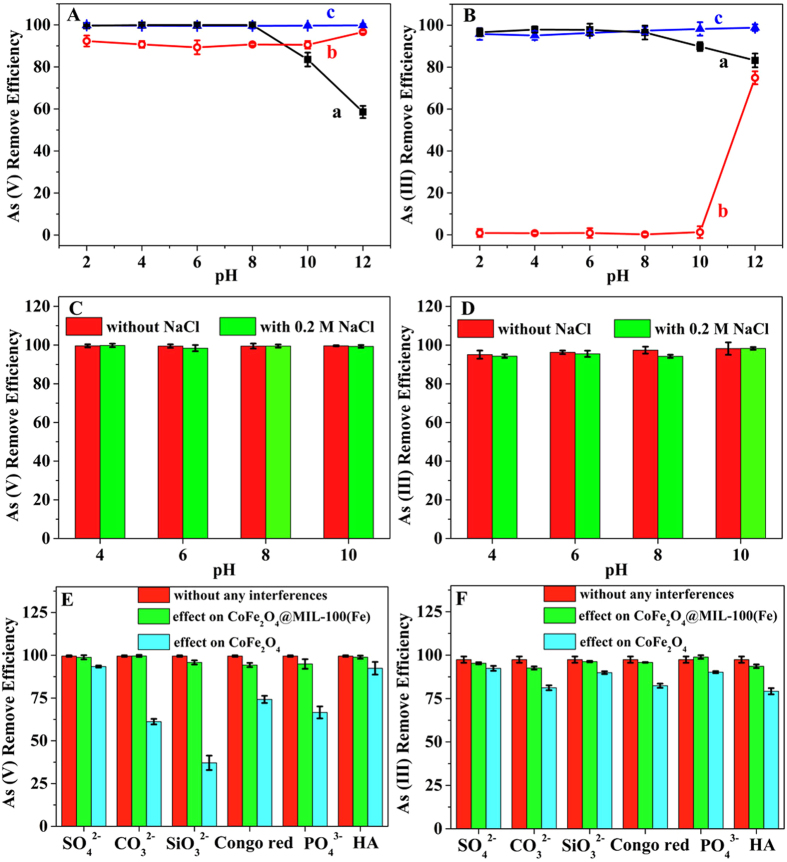
Effect of pH, ionic strength and interferences on iAs adsorption on the hybrid adsorbent. Effect of pH on the adsorption of (**A**) 1 mg L^−1^ As(V) and (**B**) 1 mg L^−1^ As(III) on (a) CoFe_2_O_4_, (b) MIL-100(Fe), and (c) CoFe_2_O_4_@MIL-100(Fe). Effect of ionic strength (0.2 M NaCl) on the adsorption of (**C**) 1 mg L^−1^ As(V) and (**D**) 1 mg L^−1^ As(III) on CoFe_2_O_4_@MIL-100(Fe) at different pH levels. Interference from co-existing species (1 mM SO_4_^2−^, 1 mM CO_3_^2−^, 1 mM SiO_3_^2−^, 1 mM Congo red, 0.1 mM PO_4_^3−^, and 50 mg L^−1^ HA) on the adsorption of (**E**) 1 mg L^−1^ As(V) and (**F**) 1 mg L^−1^ As(III) on CoFe_2_O_4_@MIL-100(Fe) with CoFe_2_O_4_ as a comparison. The dosages of the adsorbents were 0.5 g L^−1^. The high pH- and interference-tolerance capacities of CoFe_2_O_4_@MIL-100(Fe) allow iAs removal from natural water samples without any pretreatment.

Solution pH affects both the surface charge of the adsorbents and iAs speciation during adsorption. The total iAs concentration in groundwater averaged 98.6 ± 152.2 μg L^−1^ 24. The concentration of iAs in geogenic groundwater obtained from a rural well in Shanxi, China was 470 μg L^−1^. Thus, the adsorption trends of 1 mg L^−1^ As(V) or As(III) on CoFe_2_O_4_, MIL-100(Fe), and CoFe_2_O_4_@MIL-100(Fe) in the pH range of 2–12 were tested (Fig. 3A,B). To better understand the interaction of iAs speciation available at different pH levels, the main species and the curve of iAs apparent charge *versus* pH are also illustrated in Supplementary Fig. 1 and Supplementary Table 1 46.

CoFe_2_O_4_ showed a high adsorption capacity of As(V) in the pH range of 2–8 and then decreased drastically for pH 8–12 (Fig. 3A). The adsorption decreased because of the strong electrostatic repulsion between the anionic As(V) species (H_2_AsO_4_^−^ and HAsO_4_^2−^) and negatively charged CoFe_2_O_4_ in alkaline conditions^21^. Similarly, the adsorption efficiency of 1 mg L^−1^ As(III) on CoFe_2_O_4_ was higher than 98% in a pH range from 2 to 8 and then slightly decreased with a further increase of pH. Above pH 8, the amount of negatively charged H_2_AsO_3_^−^ species increased (Supplementary Table 1), and the electrostatic repulsion with the negatively charged surface of CoFe_2_O_4_ was responsible for the decreased adsorption capacity to As(III). The adsorption efficiency of As(V) on MIL-100(Fe) was approximately 90% in the pH range of 2–10 and increased to 97% at pH 12. However, MIL-100(Fe) showed almost zero adsorption of As(III) at pH < 10 and increased to 75% at pH 12. The adsorption capacity increased greatly under strongly basic conditions (pH 12) because of the dissolved Fe^3+^ from MIL-100(Fe) forming FeAsO_4_ or FeAsO_3_^11^.

CoFe_2_O_4_@MIL-100(Fe) demonstrated the highest removal efficiency for both As(V) and As(III) under the widest pH range of 2–12 by the combination of the merits from CoFe_2_O_4_ and MIL-100(Fe) (Fig. 3A and B). Electrostatic adsorption was therefore not the main driving force involved in the adsorption of iAs because of their different surface charges at different pH levels (Supplementary Fig. 1). The feasibility and applicability of the hybrid MNPs were validated as an iAs adsorbent. The obviously different adsorptions of iAs on CoFe_2_O_4_@MIL-100(Fe) and MIL-100(Fe) provides the possibility of speciation analysis of iAs. CoFe_2_O_4_@MIL-100(Fe) can be used to determine the total iAs, while MIL-100(Fe) is used to determine the As(V) content. The difference between the two results is the content of As(III).

Soluble iron can increase iAs toxicity^9^,^23^,^24^,^25^, so the stability of the hybrid MNPs adsorbent under different pH conditions was also tested with CoFe_2_O_4_ as a comparison. The stability of the hybrid adsorbent improved significantly after coating with the MIL-100(Fe) shell, as the iron concentration in the supernatants reduced from 17.8 mg L^−1^ for CoFe_2_O_4_ to 3.6 mg L^−1^ at pH 2 (Supplementary Fig. 2). The leached iron from CoFe_2_O_4_@MIL-100(Fe) was less than 0.2 mg L^−1^ at pH levels higher than 4, indicating the high stability of the hybrid MNP adsorbent. The relatively low iron leakage did not cause iron pollution or increase the toxicity of the iAs.

Considering the efficiency of iAs removal and the leakage of iron from the hybrid adsorbent, we selected pH 4–10 as the optimal iAs adsorption conditions. Our hybrid adsorbent showed high pH-tolerance because of the decreased zero-charge point (ZCP), as discussed in the adsorption mechanism section. Different solution pH ranges correspond to different iAs adsorption mechanisms: an Fe-O-As microstructure forms through hydroxyl exchange with the deprotonated arsenate and arsenite at pH 4-10^15^,^16^,^17^,^18^, while dissolved iron (Fe^3+^) is related to the formation of FeAsO_4_ or FeAsO_3_ at high or low pH^11^. Because most of natural water samples are in the pH range of 4–10, pH pre-adjustment is not required when our hybrid adsorbent is used to remove iAs from natural water samples, while the high adsorption efficiency is also retained.

### Effects of ionic strength, competing anions, and interferences on iAs adsorption

Various metal salts and ions may exist in iAs-contaminated natural water samples. Thus, it is necessary to study the effect of ionic strength on iAs adsorption. No significant change of the adsorption efficiency was observed in the presence of NaCl with concentrations up to 0.2 M in pH 4–10 (Fig. 3C and D), suggesting that the iAs adsorption on CoFe_2_O_4_@MIL-100(Fe) followed the inner-sphere complex mechanism^47^. The surface electrostatic interaction gave a negligible influence on iAs adsorption on the hybrid adsorbent. Thus, the adjustment of the ionic strength of high-salinity water samples is not required with our MNPs adsorbent for iAs removal.

Anions and biomolecules influence iAs adsorption from the As-contaminated natural water samples on some adsorbents, as reported previously^48^,^49^. Thus, 1 mM of SO_4_^2−^, CO_3_^2−^, SiO_3_^2−^, Congo red, 0.1 mM PO_4_^3−^, and 50 mg L^−1^ humic acid (HA) were selected to evaluate the effects on the removal efficiency of iAs on CoFe_2_O_4_@MIL-100(Fe) with CoFe_2_O_4_ as a comparison (the concentrations of these species are much higher than those found in natural water systems). The anti-interference capacity of the hybrid adsorbent for iAs adsorption was obviously improved after coating with MIL-100(Fe) because of the electrostatic repulsion interactions between those anions and the negatively charged MIL-100(Fe) shell (Fig. 3E and F). Macromolecules, such as Congo red and HA, did not affect the adsorption of iAs because of the size exclusion effect of the crystalline MIL-100(Fe) with the 1-nm pores^50^. Thus, the MIL-100(Fe) shell also provided the possibility of improving selectivity by its electrostatic repulsion interactions and size exclusion effect.

### Adsorption kinetics of iAs on the hybrid adsorbent

**Figure 4:**
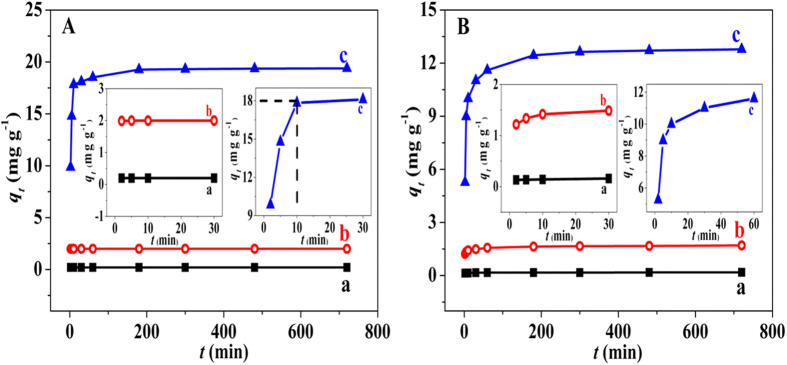
Adsorption kinetics of iAs on the hybrid adsorbent. Time-dependent adsorption of (**A**) As(V) and (**B**) As(III) on 0.5 g L^−1^ CoFe_2_O_4_@MIL-100(Fe) at different initial concentrations: (a) 0.1, (b) 1, and (c) 10 mg L^−1^. Insets: high-resolution adsorption kinetics for the first 30 or 60 min. The hybrid adsorbent exhibits a high removal efficiency and rapid uptake rate for iAs.

The time-dependent adsorption of iAs on CoFe_2_O_4_@MIL-100(Fe) was tested at three initial concentrations (0.1, 1, and 10 mg L^−1^) (Fig. 4A and B). The adsorption equilibriums of As(V) and As(III) were reached within 2 min at the 0.1 mg L^−1^ level. The residual iAs was 0.5 μg L^−1^ for As(V) and 3.9 μg L^−1^ for As(III), which was below the WHO limited value (10 μg L^−1^) in drinking water. Albeit a long period of time was required to reach adsorption equilibriums at the 1 or 10 mg L^−1^ level; the removal efficiency of iAs was higher than 95% within 10 min for As(V) and 60 min for As(III) The equilibrium adsorption of As(V) is faster than that of As(III), and our hybrid MNP adsorbent exhibited faster kinetics than most previously studied adsorbents^20^,^51^,^52^. The nanoscale size and fine particles of the MNPs, which are favorable for iAs diffusion from the bulk solution to the active adsorption sites, are responsible for the fast adsorption rate of iAs. Correspondingly, the mesoporous property is related to the high adsorption capacity by the high surface area and pore volume. Thus, the rapid uptake rate and high removal efficiency of iAs were the merits of the CoFe_2_O_4_@MIL-100(Fe) hybrid material for practical iAs removal.

To better understand the adsorption dynamics, the experimental data was analyzed with pseudo-first-order and -second-order kinetic models (Table 1). The results demonstrate that the pseudo-second-order kinetic model fitted better for both As(V) and As(III) (Supplementary Fig. 3). The calculated adsorbed amounts of iAs [*q*_*e(cal*)_] were in good agreement with the experimental results. Thus, chemisorption occurred during the iAs removal process. Valence forces through the sharing or exchange of electrons were involved between iAs and the hybrid MNPs, which agreed well with previous reports about the formation of the Fe-O-As structure^39^,^53^.

### Adsorption isotherms of iAs on the hybrid adsorbent

**Figure 5:**
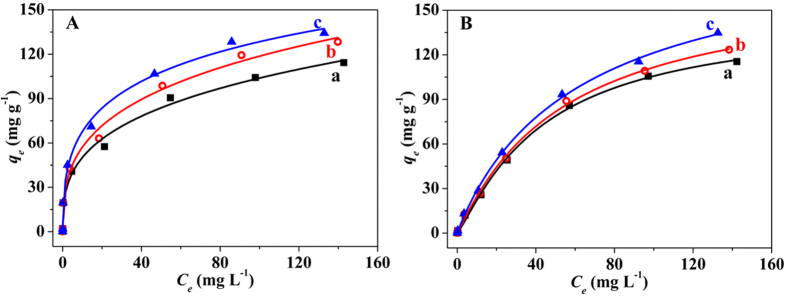
Adsorption isotherms of iAs on the hybrid adsorbent. Adsorption isotherms for the adsorption of (**A**) As(V) and (**B**) As(III) on 0.5 g L^−1^ CoFe_2_O_4_@MIL-100(Fe) at different temperatures: (a) 25, (b) 40, and (c) 50 °C.

The adsorption isotherms of As(V) and As(III) on CoFe_2_O_4_@MIL-100(Fe) were tested at three different temperatures (25, 40, and 50 °C) in the concentration range of 0.1–200 mg L^−1^ (Fig. 5A and B). The adsorption capacities of As(V) and As(III) increased with their initial concentrations, showing favorable adsorption at high concentrations of iAs. Both Langmuir and Freundlich models were utilized to fit the adsorption isotherms and propose the adsorption mechanisms (Supplementary Fig. 4). The fitting parameters of the two models at different temperatures are summarized in Table 2. The Langmuir model was more appropriate to represent the adsorption of As(V) on CoFe_2_O_4_@MIL-100(Fe). Thus, the adsorption process of As(V) was more likely a homogeneous monolayer adsorption. However, the Freundlich isotherm model closely fit the data for As(III), and the multilayer adsorption of As(III) was manifested to form the heterogeneous interface^28^. As(V) is present mainly as negatively charged H_2_AsO_4_^−^ and HAsO_4_^2−^ species at pH 7, while As(III) is fully protonated as H_3_AsO_3_ (Supplementary Fig. 1 and Supplementary Table 1). Hydroxyl (OH^−^) exchange with the negatively charged H_2_AsO_4_^−^ and HAsO_4_^2−^ species achieves the efficient adsorption of As(V). However, strong electrostatic repulsion of HAsO_4_^2−^ leads to the monolayer adsorption of As(V) to form a homogeneous surface layer. Although the adsorption of As(III) occurs with the same hydroxyl exchange procedure, the hydrogen bonds between the natural H_3_AsO_3_ molecules form a multilayer structure on the hybrid adsorbent surface. The reactions and procedures involved in the adsorption process are discussed in the following section.

The maximum adsorption capacities (*q*_*max*_) of As(V) and As(III) on the hybrid adsorbent were 114.8 and 143.6 mg g^−1^ at 25 °C (Table 2), higher than the values of most previously studied adsorbents (Supplementary Table 2). The nanoscale size and mesoporous properties of CoFe_2_O_4_@MIL-100(Fe) MNPs result in a fast adsorption rate and high adsorption capacity. Furthermore, the monolayer adsorption saturation capacity of As(III) on this hybrid adsorbent was 5.1 mg g^−1^ (please note that details about the calculation process can be found in the Supplementary Information), lower than the value of 114.8 mg g^−1^ of As(V). Thus, As(V) was more favorably adsorbed on the hybrid MNPs than As(III) because of its stronger interaction. However, the adsorption capacity of As(V) was lower than that of As(III) because of the multilayer adsorption of As(III).

To validate our hypothesis, 5 mg of CoFe_2_O_4_@MIL-100(Fe) after saturated adsorption of As(V) or As(III) was added to 5 mL of ultrapure water. After ultrasound treatment and high speed centrifugation, the concentration of As(III) in the supernatant was 22.3 mg L^−1^, much higher than the value of 1.1 mg L^−1^ of As(V). The weakly adsorbed As(III) was therefore released easily from the hybrid MNPs absorbent. The results confirmed that some adsorbents showed low As(III) adsorption capacity on the solid adsorbent because of their low surface area^26^,^27^,^28^,^29^,^30^. However, As(III) could be efficiently reserved in the mesoporous structure of our hybrid adsorbent through hydrogen bond interactions for its high adsorption capacity. The result also validated the fact that magnetic separation is relatively mild for adsorbent collection.

### Adsorption thermodynamics of iAs on the hybrid adsorbent

Thermodynamic parameters were calculated to explore the adsorption process. *K*_*0*_ (thermodynamic equilibrium constant) was calculated by plotting ln(*q*_*e*_/*C*_*e*_) (*C*_*e*_, equilibrium arsenic concentration) *versus* the amount of arsenic adsorbed (*q*_*e*_) and then extrapolating *q*_*e*_ to zero (Supplementary Fig. 5A and C). *ΔH* and *ΔS* were obtained from the slope and intercept of the plot of ln*K*_*0*_ against 1/*T*, respectively (Supplementary Fig. 5B and D). The calculated results of *ΔG, ΔH*, and *ΔS* are summarized in Table 3. The negative value of *ΔG* confirms the spontaneous nature of the adsorption process for both As(V) and As(III). The positive *ΔH* indicates an endothermic process for the adsorption of iAs on CoFe_2_O_4_@MIL-100(Fe), which is also supported by the increased adsorption capacity of iAs at high temperatures. The positive *ΔS* illustrates the increased disorder at the solid-liquid interface of CoFe_2_O_4_@MIL-100(Fe) after the adsorption of iAs, as the number of adsorbed water molecules was larger than that of the adsorbed iAs species^44^. Therefore, the adsorption of iAs on CoFe_2_O_4_@MIL-100(Fe) was dominantly controlled by an entropy-driven process.

### Adsorption mechanism of iAs on the hybrid absorbent

**Figure 6:**
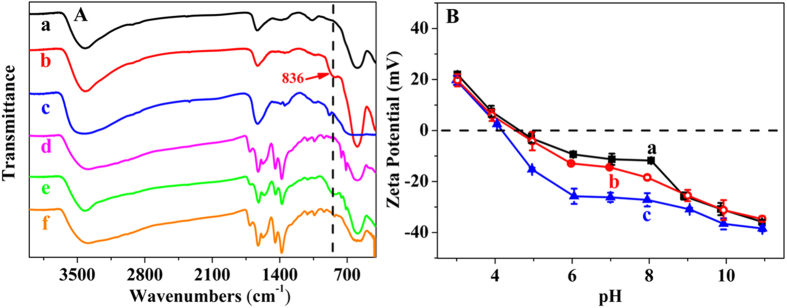
Adsorption mechanism study of iAs on the hybrid absorbent. (**A**) FTIR spectra of (a) CoFe_2_O_4_, CoFe_2_O_4_ after (b) As(V) and (c) As(III) adsorption, (d) CoFe_2_O_4_@MIL-100(Fe), CoFe_2_O_4_@MIL-100(Fe) after (e) As(V) and (f) As(III) adsorption (10 mL of 100 mg L^−1^ iAs treated with 5 mg of the adsorbent). The peak at approximately 836 cm^−1^ was assigned to the stretching vibration of the Fe-O-As group. (**B**) Zeta potentials of (a) CoFe_2_O_4_@MIL-100(Fe) and CoFe_2_O_4_@MIL-100(Fe) after (b) As(III) and (c) As(V) adsorption (10 mL of 100 mg L^−1^ iAs treated with 5 mg of adsorbents). The zero charge point of the hybrid adsorbent was 4.7. The zeta potentials of CoFe_2_O_4_@MIL-100(Fe) decreased after iAs adsorption, suggesting the formation of negatively charged inner-sphere complexes between iAs and the hybrid adsorbent.

**Figure 7:**
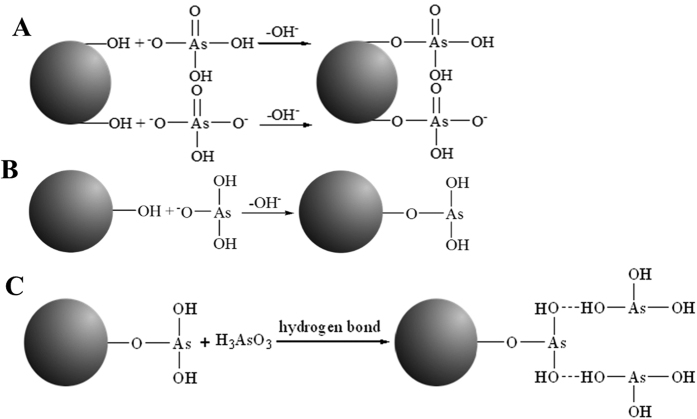
The structure of iAs on the hybrid absorbent. (**A**,**B**) The substitution of −OH groups on CoFe_2_O_4_@MIL-100(Fe) by the deprotonated iAs species through the hydroxyl exchange procedure. (**C**) Natural H_3_AsO_3_ was adsorbed on the hybrid adsorbent through hydrogen bonding to form a multilayer structure for the high adsorption capacity of As(III).

FTIR spectra and zeta potentials reveal the structure of iAs adsorbed on CoFe_2_O_4_@MIL-100(Fe). The peak at approximately 836 cm^−1^ was assigned to the stretching vibration of the Fe-O-As group (Fig. 6A). Therefore, CoFe_2_O_4_ and CoFe_2_O_4_@MIL-100(Fe) have the same adsorption mechanism toward As(V) and As(III), with monodentate attachment of deprotonated arsenate and arsenite, which agrees well with the X-ray absorption spectroscopic (XAS) data^33^. The -OH groups on the CoFe_2_O_4_@MIL-100(Fe) surface were substituted by the deprotonated iAs species through the hydroxyl exchange procedure, as illustrated in Fig. 7A and B 49.

The adsorption effectiveness and efficiency of iAs was therefore controlled by the distribution coefficient of deprotonated iAs species. Equations 1,2,3 represent the three-order dissociation constants of As(V), which illustrate the deprotonation procedure. At pH 7, almost all As(V) exists in the forms of H_2_AsO_4_^−^ or HAsO_4_^2−^ (Eq. 4). Thus, As(V) is more favorably adsorbed on the hybrid MNPs through the hydroxyl exchange with H_2_AsO_4_^−^ and HAsO_4_^2−^ 54. However, H_3_AsO_3_ has a low dissociation constant of 6.0 × 10^−10^ (H_3_AsO_3_ is a tribasic weak acid, and the other two deprotonation procedures are ignored, as validated by the result in Supplementary Table 1). The distribution coefficient of H_2_AsO_3_^−^ is only 0.006 (Eqs 5 and 6). Thus, As(III) shows a lower hydroxyl exchange efficiency than As(V). Furthermore, natural H_3_AsO_3_ was adsorbed on the adsorbent through hydrogen bonding to form a multilayer structure for its high adsorption capacity (Fig. 7C).


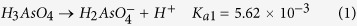



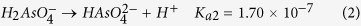



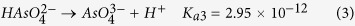



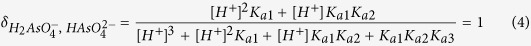



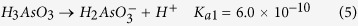



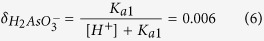


The above adsorption mechanism was also confirmed by the zeta potentials of CoFe_2_O_4_@MIL-100(Fe) before and after the adsorption of As(V) and As(III) (Fig. 6B). The zeta potentials of CoFe_2_O_4_@MIL-100(Fe) decreased obviously along with increased solution pH. The surfaces of the CoFe_2_O_4_ nanoparticles were covered with hydroxyl groups. Therefore, the surface charge changes as the different groups are changed from -FeOH_2_^+^, -FeOH, to -Fe(OH)_2_^−^ and even -Fe(OH)_3_^2−^ along with the increased pH. The ZCP, the pH of zero charge of an absorbent, therefore becomes a critical point for the iAs adsorption through hydroxyl exchange. Interestingly, the ZCP of CoFe_2_O_4_@MIL-100(Fe) is 4.7, obviously lower than “bare” CoFe_2_O_4_ MNPs adsorbent, whose ZCP was approximately eight^21^. Thus, our adsorbent facilitates iAs adsorption by its abundant hydroxyl groups as the adsorbed sites and a low ZCP. The ZCP result also validated the wide pH range of our hybrid adsorbent for iAs adsorption.

After As(V) adsorption, the zeta potentials of CoFe_2_O_4_@MIL-100(Fe) decreased rapidly as direct evidence of the strong specific adsorption and inner-sphere complexes formed on the surface of the hybrid absorbent^21^. However, almost the same zeta potential trend was observed after adsorbing As(III). The inner-complex formation could also keep a constant surface charge, as shown in Fig. 7B for As(III) adsorption. Thus, both As(V) and As(III) form inner-sphere complexes with the hybrid adsorbent, as verified by the FTIR results; the negative charge prohibits the further adsorption of As(V). Multilayer adsorption of As(III) occurs through hydrogen bonds, as shown in Fig. 7C. As(V) is therefore more strongly adsorbed than As(III), but a higher adsorption capacity was observed for As(III). Due to the mesoporous property of our hybrid adsorbent, the adsorbed As(III) was embedded in the pores for its highly efficient and stable adsorption capacity.

### Applicability of CoFe_2_O_4_@MIL-100(Fe) to iAs-contaminated water samples

**Figure 8:**
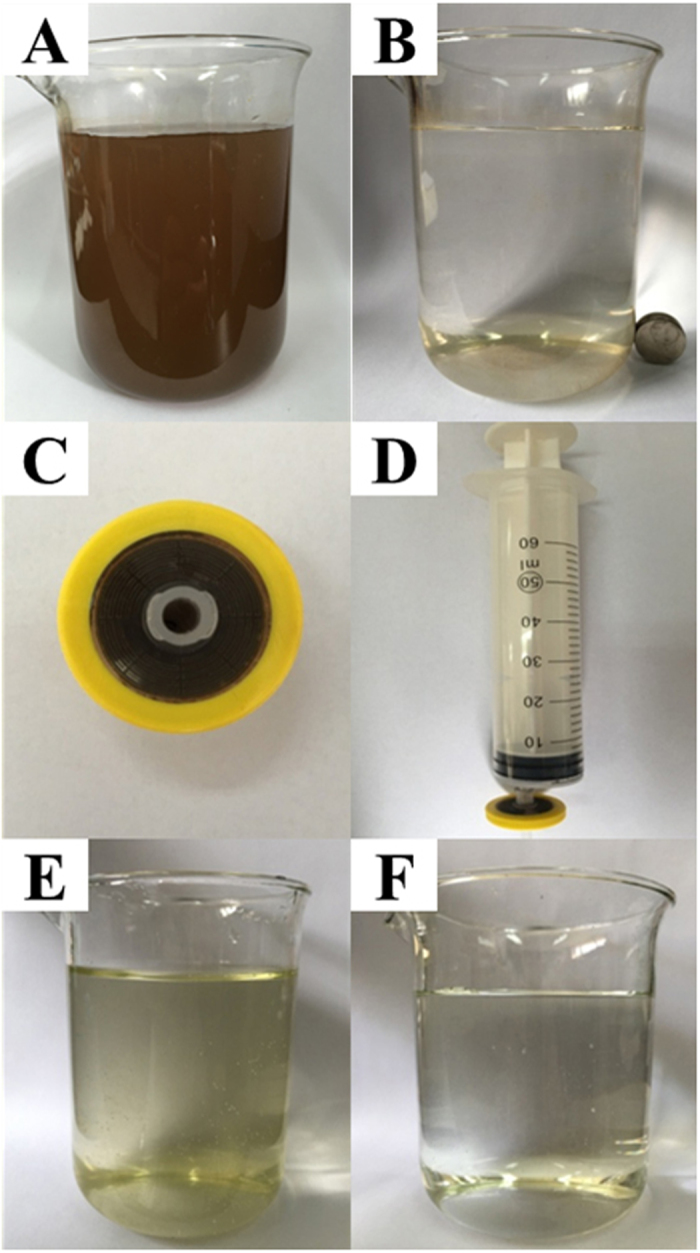
Two kinds of simple water treatment strategies. (**A**,**B**) Batch adsorption of iAs from 500 mL of a sample of well water with a high arsenic concentration (470 μg L^−1^ iAs) with 1 g of CoFe_2_O_4_@MIL-100(Fe). The hybrid adsorbent was recovered simply with a magnet after iAs adsorption. (**C**,**D**) The photos of a simple water filter and (**E**,**F**) the photos of a water sample before and after treatment with the water filter. The operational convenience of the filtration strategy allows the removal of iAs and solid particles simultaneously.

Batch treatment of iAs-contaminated groundwater sample from a rural well in Shanxi, China was first used to evaluate the practicability of CoFe_2_O_4_@MIL-100(Fe) to remove iAs (Fig. 8A and B). The removal of iAs from 500 mL of highly iAs-contaminated water (470 μg L^−1^) down to WHO drinking water standard could be achieved by 1 g of the hybrid adsorbent with a final iAs concentration of 4.2 μg L^−1^. The hybrid adsorbent can be simply recovered by a magnet after iAs adsorption (Fig. 8B). Batch treatment mode with CoFe_2_O_4_@MIL-100(Fe) MNPs is very suitable for processing large quantities of iAs-contaminated natural water.

To improve the operational convenience for practical application, a simple water filter system was fabricated by packing 10 mg of CoFe_2_O_4_@MIL-100(Fe) MNPs in a commercial filter (Fig. 8C and D). This filter was used for the treatment of 100 mL of iAs-containing simulated water samples. The concentration of iAs decreased from 100 μg L^−1^ [80 μg L^−1^ As(V) and 20 μg L^−1^ As(III)] to 4.1 μg L^−1^ in the filtrate, with the final concentration lower than the WHO standard of 10 μg L^−1^. Moreover, the simple water filtration strategy can also remove particle impurities in water sample simultaneously. The turbidity of the iAs-containing water sample decreased from 4.9 to 1.2 NTU, as calculated by the spectrophotometric method (Fig. 8E and F). This simple filtration strategy is ideal for the on-site treatment of iAs-contaminated water in nature. Its great portability and excellent purification efficiency makes the filter competitive among treatment systems available for iAs removal as a small and simple water facility.

### Speciation analysis of iAs in real samples

In this work, we found that MIL-100(Fe) showed favorable adsorption of As(V), while CoFe_2_O_4_@MIL-100(Fe) had a high adsorption capacity for both As(V) and As(III) in the pH range of 4–10. Therefore, we tried to use MIL-100(Fe) and CoFe_2_O_4_@MIL-100(Fe) for the speciation analysis of iAs in natural water samples. MIL-100(Fe) was used to adsorb As(V), and the hybrid MNPs adsorbent was used to determine the total iAs. The concentration of As(III) was obtained by the difference between total iAs and As(V). The accuracy of this arsenic speciation strategy was checked by analyzing two natural water samples (Table 4). In total, 338.8 μg L^−1^ of As(III) and 126.7 μg L^−1^ of As(V) were found in the first water sample, which was obtained from Shanxi, China. The result was consistent with the total As concentration of 470 μg L^−1^. High As(III) content showed the typical property of hypoxic As-contaminated groundwater, which was collected from a rural well in Shanxi, China. The content of iAs in the second sample was below the detection limits of the proposed method. The recovery results demonstrate the feasibility of the proposed method for practical applications.

### Summary

In summary, we have developed core-shell CoFe_2_O_4_@MIL-100(Fe) hybrid magnetic nanoparticles (MNPs) as a nanoadsorbent for iAs removal. The nanoscale size and mesoporous structure of the hybrid adsorbent exhibits excellent adsorption performance, such as fast adsorption kinetics and a high adsorption capacity. The MIL-100(Fe) shell not only improved the anti-interference capacity with the electrostatic repulsion and size exclusion effects but also decreased the ZCP of the hybrid adsorbent to 4.7, obviously lower than that of “bare” CoFe_2_O_4_ MNPs, whose ZCP is approximately 8. Therefore, a wide pH range of 4–10 was obtained for iAs adsorption. Based on the adsorption kinetics, isotherms, and thermodynamics of iAs on the hybrid adsorbent, the adsorption mechanism was proposed. The good stability, fast adsorption rate, high adsorption capacity, excellent pH and interference-tolerance capacities of CoFe_2_O_4_@MIL-100(Fe) were validated by two kinds of simple water treatment strategies: a batch magnetic separation mode and a simple filtration strategy for the simultaneous removal of particle impurities and iAs. MIL-100(Fe) and the CoFe_2_O_4_@MIL-100(Fe) hybrid adsorbent were used to successfully achieve speciation analysis of iAs in water samples from natural sources.

## Methods

### Materials

All reagents used in the experiment were of analytical grade. 1, 3, 5-Benzenetricarboxylic acid (H_3_BTC), FeCl_3_·6H_2_O and CoCl_2_·6H_2_O were purchased from Bodi Chemical Reagent Co., Ltd., Tianjin, China. Mercaptoacetic acid (MAA), ethylene glycol (HOCH_2_CH_2_OH), polyethylene glycol (PEG-6000), nitric acid (HNO_3_), hydrofluoric acid (HF) and humic acid (HA) were purchased from Fuchen Chemical Reagent Co., Ltd., Tianjin, China. 1000 mgL^−1^ of As(V) and As(III) stock solutions were obtained from Sigma-Aldrich Co., Ltd., Shanghai, China.

### Apparatus

Transmission electron microscopy (TEM) images were obtained from JEM-2010HR microscope. Fourier transform infrared spectra (FT-IR) were recorded on a Fourier transform infrared spectrometer. Thermogravimetric analysis (TGA) of the adsorbents was carried out on a PTC-10ATG-DTA analyzer heated from room temperature to 700 °C with an air flow rate of 100 mL min^−1^. The magnetic properties of the adsorbents were measured at room temperature under a varying magnetic field from −10 k to 10 k Oe. X-ray diffraction (XRD) measurements were performed on a D/max-2500 diffractometer using Cu-Kα radiation (λ = 1.5418 Å). The surface area and pore size distribution of adsorbents were studied by N_2_ adsorption-desorption isotherms at 77 K. The hydrodynamic sizes and zeta potentials of the adsorbents were measured using a Zetasizer Nano ZS instrument. The concentrations of iAs were measured by an inductively coupled plasma mass spectrometer (ICP-MS).

### The preparation of CoFe_2_O_4_

Mesoporous CoFe_2_O_4_ magnetic nanoparticles (MNPs) were synthesized by a facile one-pot hydrothermal reaction according to a previous report with slight modification^55^. 148.7 mg CoCl_2_·6H_2_O, 337.9 mg FeCl_3_·6H_2_O, 900.0 mg CH_3_COONa·3H_2_O, and 500.0 mg PEG-6000 were dissolved in 10.0 mL HOCH_2_CH_2_OH under stirring at 50 °C for 10 min to form a homogeneous solution. The mixture was heated at 160 °C for 16 h. The black product was magnetically separated and rinsed with deionized water. Finally, the obtained CoFe_2_O_4_ were dried at 60 °C for 6 h.

### The preparation of CoFe_2_O_4_@MIL-100(Fe)

CoFe_2_O_4_@MIL-100(Fe) MNPs was fabricated by a simple step-by-step self-assembly strategy as previously reported with some modification^45^,^56^,^57^. CoFe_2_O_4_ were first modified by MAA, which was added in 10 mL ethanol containing 50 mg CoFe_2_O_4_. The concentration of MAA was maintained 0.58 mM. The mixture solution was stirred for 24 h at room temperature under the protection of nitrogen to avoid the oxidation of MAA. Then the obtained product was separated and washed with ethanol to remove excess MAA. MAA-modified CoFe_2_O_4_ were alternately immersed in 4 mL of 50 mM FeCl_3_·6H_2_O ethanol solution for 15 min and 4 mL of 50 mM H_3_BTC ethanol solution for 30 min at 70 °C in oil bath. Before each step, the as-synthesized samples were washed with ethanol. Finally, after 10 cycles growth, the fabricated CoFe_2_O_4_@MIL-100(Fe) MNPs were dried at 90 °C under vacuum.

### The preparation of MIL-100(Fe)

MIL-100(Fe) was prepared according to Yoon *et al*. report^58^. Briefly, 138.5 mg iron powder, 343.5 mg H_3_BTC, 100 mL HF (35%) and 95 mL HNO_3_ (65%) were mixed well in 10 mL ultrapure water. The solution was heated to 150 °C for 12 h. After cooled down to room temperature and washed with ultrapure water and ethanol, the resulting MIL-100(Fe) was dried at 80 °C under vacuum.

### General adsorption procedure

The adsorption capacity (*q*_*e*_) in the whole adsorption experiments was calculated according to the following equation:


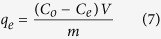


where *C*_*0*_ and *C*_*e*_ are the initial and final concentration of arsenic solution (mg L^−1^). *V* is the volume of arsenic solution (L), and *m* is the amount of adsorbent (g).

### Effect of pH on iAs adsorption

To study the effect of pH, 1 M HCl and NaOH were used to adjust the pH of the iAs solutions as required. Initial As(V) and As(III) concentrations were 1 mg L^−1^ and the dosages of the adsorbents were 0.5 g L^−1^.

The leaching behaviors of CoFe_2_O_4_ and CoFe_2_O_4_@MIL-100(Fe) were investigated to examine the stability of the adsorbents. 5 mg adsorbents were immerged into 10 mL aqueous solution with pH ranging from 2 to 12 and stirred for 24 h. Then, the concentrations of leaching iron ions in the supernatants were analyzed by ICP-MS.

### Effect of ionic strength on iAs adsorption

The effect of ionic strength was studied by dissolving certain amount of NaCl soild in 10 mL of 1 mg L^−1^ iAs solutions at different pH. The concentration of NaCl was maintained at 0.2 M.

### Effect of competing anions and interferences

1 mM Na_2_SO_4_, Na_2_CO_3_, Na_2_SiO_3_, Congo red, 0.1 mM Na_3_PO_4_, and 50 mg L^−1^HA were selected to investigate the effect of competing anions and interferences on iAs adsorption. Initial As(V) and As(III) concentrations were 1 mg L^−1^, and the reaction time was 12 h.

### Adsorption kinetics of iAs on CoFe_2_O_4_@MIL-100(Fe)

0.1, 1 and 10 mg L^−1^As(V) and As(III) solutions were used to study the adsorption kinetics at 25 °C. After adsorption for a predetermined period (from 2 to 720 min), the residual iAs in solutions were determined by ICP-MS. The experimental data of dynamics adsorption were analyzed based on eqs (8) and (9), respectively.


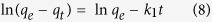



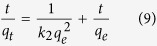


where *q*_*e*_ (mg g^−1^) and *q*_*t*_ (mg g^−1^) represent the amounts of iAs adsorbed at equilibrium and at the time *t* (min); *k*_*1*_ (min^−1^) and *k*_*2*_ (g mg^−1^ min^−1^) are the rate constants of the pseudo-first-order and pseudo-second-order kinetic models, respectively.

### Adsorption isotherms of iAs on CoFe_2_O_4_@MIL-100(Fe)

Adsorption isotherms were studied with initial concentrations of iAs varying from 0.1 to 200 mg L^−1^. The adsorption proceeded for 12 h to reach adsorption equilibrium, and the experimental temperatures were controlled at 25, 40 and 50 °C, respectively. Langmuir and Freundlich models were utilized to fit the adsorption isotherms based on eqs (10) and (11).


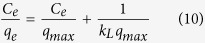



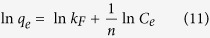


where *q*_*e*_ (mg g^−1^) is the amount of iAs adsorbed at equilibrium, *C*_*e*_ (mg L^−1^) is the equilibrium iAs concentration, *k*_*L*_ (L mg^−1^) represents the Langmuir constant involving the adsorption energy and affinity of binding sites, and *q*_*max*_ (mg g^−1^) denotes the Langmuir monolayer adsorption capacity, *k*_*F*_ (mg g^−1^) is the Freundlich constant and *n* is the heterogeneity factor related to adsorption capacity and adsorption intensity.

Monolayer adsorption saturation capacity of As(III) on CoFe_2_O_4_@MIL-100(Fe) was calculated based on Gibbs free energy change (*ΔG*) from adsorption thermodynamics. As well known, the hydrogen bond is extremely weak compared to the covalent bond. Thus, it is assumed that hydrogen bond can be ignored and *ΔG* in the adsorption process comes from monolayer adsorption of As(III) mainly. Monolayer adsorption saturation capacity of As(III) on CoFe_2_O_4_@MIL-100(Fe) was determined by the following equation:


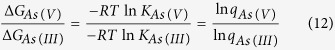


Where *ΔG*_*As(V*)_ and *ΔG*_*As(III*)_ (kJ mol^−1^) are Gibbs free energy change of iAs during adsorption, *K*_*As(V*)_ and *K*_*As(III*)_ are the thermodynamic equilibrium constant, *q*_*As(V*)_ and *q*_*As(III*)_ (mg g^−1^) are the monolayer adsorption saturation capacity of iAs on CoFe_2_O_4_@MIL-100(Fe).

### Adsorption thermodynamics of iAs on CoFe_2_O_4_@MIL-100(Fe)

The thermodynamic parameters for the adsorption processwere calculated at each temperature according to the following equations:


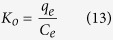



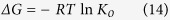



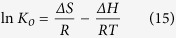


where *q*_*e*_ (mg g^−1^) is the adsorbed amount of iAs on CoFe_2_O_4_@MIL-100(Fe), *C*_*e*_ (mg L^−1^) is the equilibrium concentration of iAs, *K*_*0*_ is the thermodynamic equilibrium constant, *ΔG* (kJ mol^−1^) represents Gibbs free energy change, *ΔH* (kJ mol^−1^)is enthalpy change, *ΔS* (J mol^−1^ K^−1^) is entropy change.

### Applicability of CoFe_2_O_4_@MIL-100(Fe) to iAs contaminated water samples

Two kinds of water treatment strategies: batch mode and filtration strategy were conducted to examine the application of CoFe_2_O_4_@MIL-100(Fe) to remove iAs in real water samples.

High arsenic real water sample was collected from a rural well in Shanxi, China, and 500 mL of the water sample was treated with 1 g CoFe_2_O_4_@MIL-100(Fe). After batch adsorption, the residual iAs concent was determined.

In order to improve the operation convenience of the adsorbent for practical application, a simple water filter was prepared by packing 10 mg CoFe_2_O_4_@MIL-100(Fe) into a 0.2 μm commercial filter. 200 mL simulated water sample containing 80 μg L^−1^As(V) and 20 μg L^−1^As(III) was poured through the filter slowly with a syringe. The effluent solutions were collected at predetermined volume intervals, and the concentrations of the residual iAs in the effluents were monitored.

### Speciation analysis of As(V) and As(III) in real samples

MIL-100(Fe) and CoFe_2_O_4_@MIL-100(Fe) were used to speciation analysis of As(V) and As(III) in real water samples. Two real water samples (W1 and W2) were collected from a river in Shuangzhai village, Shanxi, China and Weijin River in Nankai zone, Tianjin, China.

The concentrations of As(V) and As(III) in W1 and W2 were determined according to the standard addition method. Firstly, 5 mg MIL-100(Fe) was added in 10 mL water samples, after adsorption of As(V) for 1 h, 1 mg MIL-100(Fe) was collected and dissolved with concentrated HNO_3_ and diluted to 5 mL for the determination of As(V). Secondly, 5 mg CoFe_2_O_4_@MIL-100(Fe) was added in another 10 mL water sample, after adsorption of both As(V) and As(III) for 1 h, 1 mg CoFe_2_O_4_@MIL-100(Fe) was treated the same as MIL-100(Fe), including HNO_3_ digestion and determination of total iAs. The concentration of As(III) was obtained by the difference between As(V) and total iAs.

## Additional Information

**How to cite this article**: Yang, J.-C. and Yin, X.-B. CoFe_2_O_4_@MIL-100(Fe) hybrid magnetic nanoparticles exhibit fast and selective adsorption of arsenic with high adsorption capacity. *Sci. Rep.* **7**, 40955; doi: 10.1038/srep40955 (2017).

**Publisher's note:** Springer Nature remains neutral with regard to jurisdictional claims in published maps and institutional affiliations.

## Supplementary Material

Supplementary Information

## Figures and Tables

**Table 1:** Kinetic model parameters for the adsorption of iAs on CoFe_2_O_4_@MIL-100(Fe).

	*C*_*0*_ (mg L^−1^)	*q*_*e(exp*)_ (mg g^−1^)	Pseudo-first-order kinetic model			Pseudo-second-order kinetic model		
			*q*_*e(cal*)_ (mg g^−1^)	*k*_*1*_ (min^−1^)	R^2^	*q*_*e(cal*)_ (mg g^−1^)	*k*_*2*_ (g mg^−1^ min^−1^)	R^2^
As(V)	0.1	0.20	5.00 × 10^−4^	4.29 × 10^−3^	0.43	0.20	212.63	1.00
	1	1.99	1.05 × 10^−3^	8.26 × 10^−3^	0.63	1.99	54.20	1.00
	10	19.36	2.58	1.07 × 10^−2^	0.82	19.47	0.02	1.00
As(III)	0.1	0.19	4.63 × 10^−3^	3.44 × 10^−2^	0.84	0.19	0.84	1.00
	1	1.93	5.50 × 10^−3^	0.29	0.85	1.89	0.13	1.00
	10	14.10	8.96 × 10^−3^	3.08	0.88	13.66	1.66 × 10^−2^	1.00

**Table 2:** Langmuir and Freundlich parameters for the desorption of iAs on CoFe_2_O_4_@MIL-100(Fe).

Langmuir model					Freundlich model		
	*T* (°C)	*q*_*max*_ (mg g^−1^)	*k*_*L*_ (L mg^−1^)	R^2^	*k*_*F*_ (mg g^−1^)	*n*	R^2^
As(V)	25	114.8	0.14	0.98	12.80	1.95	0.88
	40	129.9	0.14	0.98	14.27	1.91	0.88
	50	136.1	0.19	0.99	16.06	1.89	0.87
As(III)	25	143.6	2.60 × 10^−2^	0.93	3.51	1.29	0.98
	40	153.0	2.65 × 10^−2^	0.93	3.77	1.28	0.99
	50	167.5	2.69 × 10^−2^	0.93	4.18	1.29	0.99

**Table 3:** Thermodynamic parameters for adsorption of iAs on CoFe_2_O_4_@MIL-100(Fe).

	*T* (°C)	Thermodynamic parameters			
		ln *K*_*0*_	*Δ G* (kJ mol^−1^)	*Δ H* (kJ mol^−1^)	*Δ S* (J mol^−1^ K^−1^)
As(V)	25	3.12	−7.73	27.73	118.50
	40	3.48	−9.06		
	50	4.01	−10.76		
As(III)	25	1.07	−2.65	7.47	33.90
	40	1.19	−3.10		
	50	1.31	−3.52		

**Table 4:** Analytical results for the arsenic species in real samples.

As(III)				As(V)		
Samples	Add (μg L^−1^)	Found^a^ (μg L^−1^)	Recovery (%)	Add (μg L^−1^)	Found^a^ (μg L^−1^)	Recovery (%)
W1	0	338.8 ± 4.2	—	0	126.7 ± 5.2	—
	100	451.6 ± 8.7	112.8	100	213.8 ± 3.6	87.1
	200	518.6 ± 6.7	89.9	200	320.4 ± 10.4	96.9
W2	0	0	—	0	0	—
	100	96.4 ± 8.3	96.4	100	104.7 ± 4.3	104.7
	200	202.3 ± 3.5	101.2	200	185.4 ± 12.1	92.7

^a^Mean of three measurements.
