# Supplementary material for: CoFe2O4@MIL-100(Fe) hybrid magnetic nanoparticles exhibit fast and selective adsorption of arsenic with high adsorption capacity
Source: Sci Rep. 2017 Jan 19;7:40955. doi: 10.1038/srep40955 (PMC5244426; doi:10.1038/srep40955)
Supplement: Supplementary Information [file srep40955-s1.docx]

**Supplementary Information for**

**CoFe_2_O_4_@MIL-100(Fe) hybrid magnetic nanoparticles exhibit fast and selective adsorption of arsenic with high adsorption capacity**

Ji-Chun Yang^1^, Xue-Bo Yin*^,1,2^

^1^State Key Laboratory of Medicinal Chemical Biology andTianjin Key Laboratory of Biosensing and Molecular Recognition, College of Chemistry, Nankai University, Tianjin, 300071, China

^2^ Collaborative Innovation Center of Chemical Science and Engineering (Tianjin), Nankai University, Tianjin, 300071, China

* E-mail: xbyin@nankai.edu.cn; Fax: (+86) 022-23503034

1. **Results and Discussion**





**Supplementary Figure 1 | Apparent charges of (a) As(V) and (b) As(III) *versus* pH.**

**Supplementary Table 1 | The species of iAsat different pH**

| pH | < 2.3 | 2.3-6.9 | | 6.9-11 | > 11 |
| --- | --- | --- | --- | --- | --- |
| As(V) speciation | H_3_AsO_4_ | H_2_AsO_4_^-^ | | HAsO_4_^2-^ | AsO_4_^3-^ |
| pH | <9.0 | | 9-12 | 13 | 14 |
| As(III) speciation | H_3_AsO_3_ | | H_2_AsO_3_^-^ | HAsO_3_^2-^ | AsO_3_^3-^ |

**

**

**Supplementary Figure 2 | The stability of CoFe_2_O_4_ and CoFe_2_O_4_@MIL-100(Fe).** Iron ion concentrations leached out from CoFe_2_O_4_ (a) and CoFe_2_O_4_@MIL-100(Fe) (b) with pH values ranging from 2 to 12. The stability of the hybrid adsorbent improved significantly after coated with MIL-100(Fe) shell.


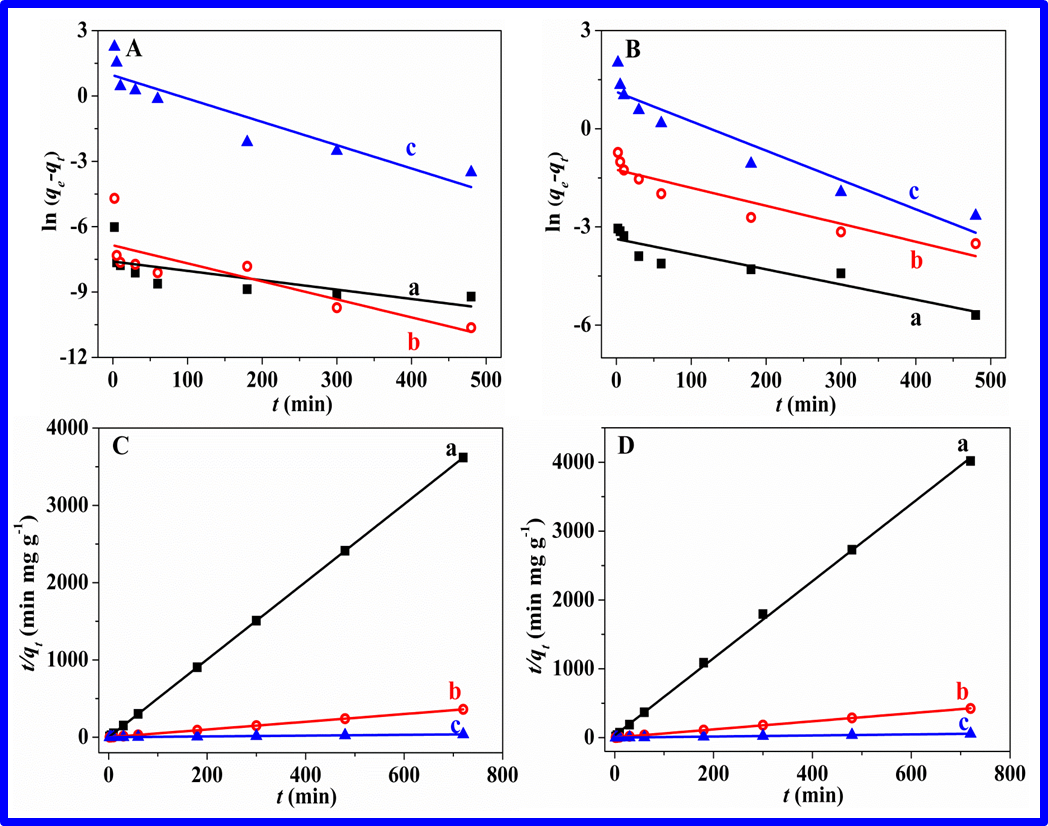


**Supplementary Figure 3 |Adsorption kinetics of iAs on the hybrid adsorbentanalyzed with pseudo-first-order and –second-order kinetic models.** The plots of pseudo-first-order kinetics (A, B) and pseudo-second-order kinetics (C, D) for the adsorption of As(V) (A, C) and As(III) (B, D) on 0.5 g L^-1^ CoFe_2_O_4_@MIL-100(Fe) at different initial concentrations: 0.1 mg L^-1^ (a), 1 mg L^-1^ (b) and 10 mg L^-1^ (c).Pseudo-second-order kinetic model fits better than pseudo-first-order one for both As(V) and As(III).


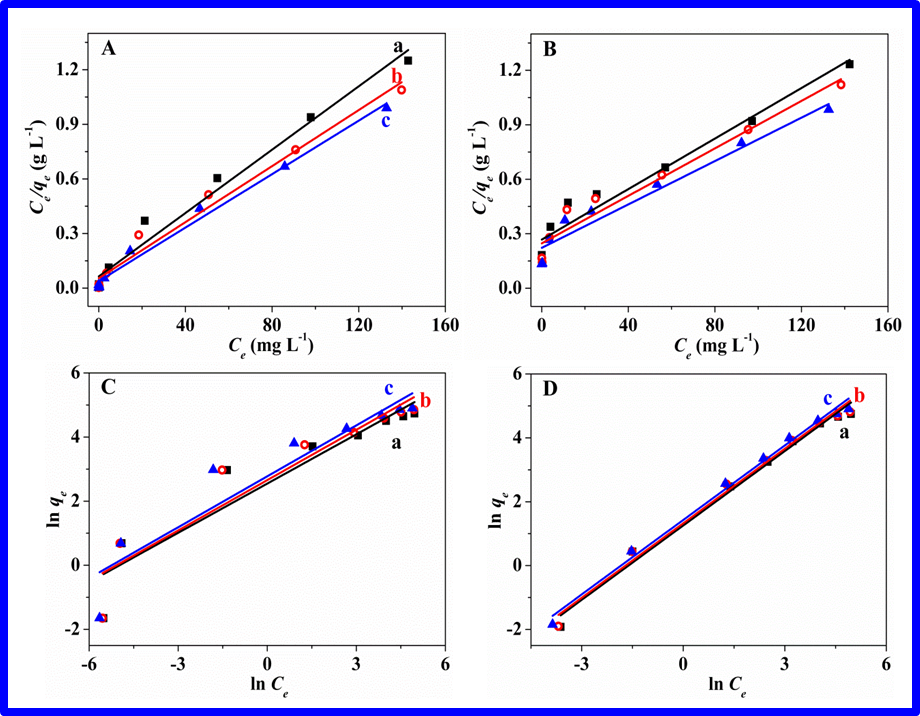


**Supplementary Figure 4 |Adsorption isotherms of iAs on the hybrid adsorbent analyzed with Langmuir and Freundlich models.** The corresponding Langmuir (A, B) and Freundlich (C, D) plots for the adsorption of As(V) (A, C) and As(III) (B, D) on 0.5 g L^-1^ CoFe_2_O_4_@MIL-100(Fe) at different temperature: 25 °C (a), 40 °C (b), and 50 °C (c). Langmuir model was more appropriate to represent the adsorption isotherm of As(V) on CoFe_2_O_4_@MIL-100(Fe), while better regression coeﬃcient was observed with Freundlich isotherm model for adsorption of As(III).

**Supplementary Table 2 |Comparison of adsorption capacities of iAs on various adsorbents**

| adsorbent | pH | adsorption capacity  (mg g^-1^) | | reference |
| --- | --- | --- | --- | --- |
|  |  | As(V) | As(III) |  |
| Fe_3_O_4_ | 7 | 44.1 | 49.8 | ^1^ |
| MnFe_2_O_4_ | 7 | 90.4 | 93.8 | ^1^ |
| CoFe_2_O_4_ | 7 | 73.8 | 100.3 | ^1^ |
| goethite | 5 | 5 | — | ^2^ |
| Fe-Ti composite | 7 | 14.3 | 85 | ^3^ |
| MWNT/Fe_3_O_4_ hybrid | 7 | 53 | 39 | ^4^ |
| graphene-α-FeOOH aerogel | 8-9 | 81.3 | 13.4 | ^5^ |
| Fe_3_O_4_-graphene composites | 7 | 0.4 | 0.3 | ^6^ |
| CoFe_2_O_4_@MIL-100(Fe) | 4-10 | 114.8 | 143.6 | This work |


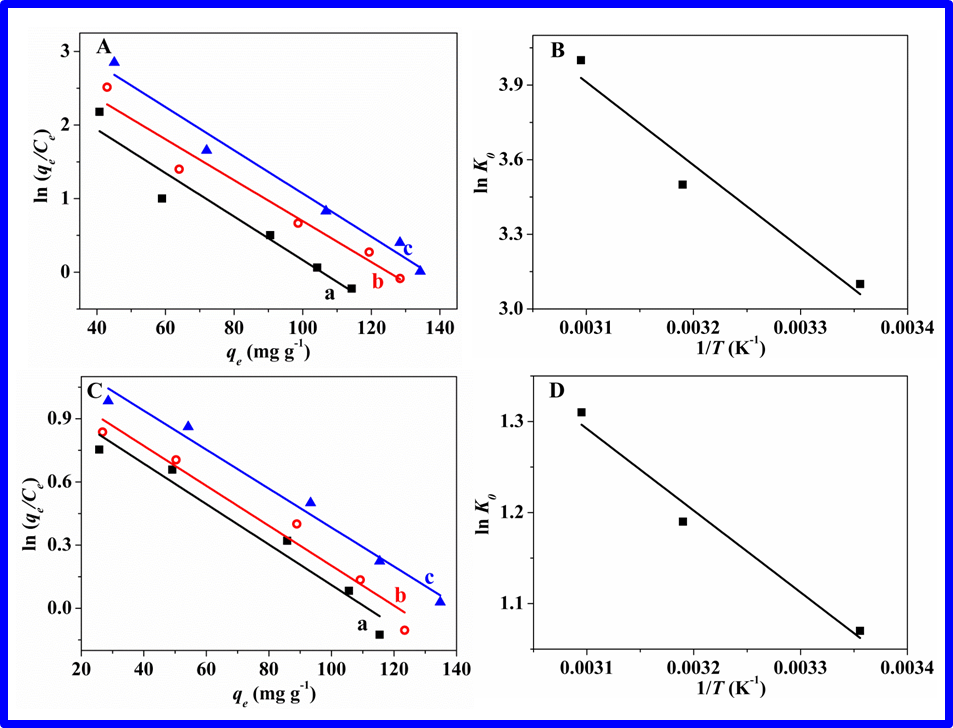


**Supplementary Figure 5 |Adsorption thermodynamics of iAs on the hybrid adsorbent.** Dependence of ln (*q_e_/C_e_*) and *q_e_* for the adsorption of As(V) (A, B) and As(III) (C, D) on 0.5 g L^-1^ CoFe_2_O_4_@MIL-100(Fe) at (A, C) different temperatures and (B, D) ln *K_0_* against1/*T*.

1. **Supplementary References**

1 Zhang, S., Niu, H., Cai, Y., Zhao, X. & Shi, Y. Arsenite and arsenate adsorption on coprecipitated bimetal oxide magnetic nanomaterials: MnFe_2_O_4_ and CoFe_2_O_4_. *Chem. Eng. J.* **158**, 599-607, doi:http://dx.doi.org/10.1016/j.cej.2010.02.013 (2010).

2 Lakshmipathiraj, P., Narasimhan, B. R. V., Prabhakar, S. & Bhaskar Raju, G. Adsorption of arsenate on synthetic goethite from aqueous solutions. *J. Hazard. Mater.* **136**, 281-287, doi:http://dx.doi.org/10.1016/j.jhazmat.2005.12.015 (2006).

3 Gupta, K. & Ghosh, U. C. Arsenic removal using hydrous nanostructure iron(III)–titanium(IV) binary mixed oxide from aqueous solution. *J. Hazard. Mater.* **161**, 884-892, doi:http://dx.doi.org/10.1016/j.jhazmat.2008.04.034 (2009).

4 Mishra, A. K. & Ramaprabhu, S. The role of functionalised multiwalled carbon nanotubes based supercapacitor for arsenic removal and desalination of sea water. *J. Exp. Nanosci.* **7**, 85-97, doi:10.1080/17458080.2010.509872 (2012).

5 Andjelkovic, I. *et al.* Graphene Aerogels Decorated with α-FeOOH Nanoparticles for Efficient Adsorption of Arsenic from Contaminated Waters. *Acs Appl. Mater. Inter.* **7**, 9758-9766, doi:10.1021/acsami.5b01624 (2015).

6 Guo, L., Ye, P., Wang, J., Fu, F. & Wu, Z. Three-dimensional Fe_3_O_4_-graphene macroscopic composites for arsenic and arsenate removal. *J. Hazard. Mater.* **298**, 28-35, doi:http://dx.doi.org/10.1016/j.jhazmat.2015.05.011 (2015).
